# Pathological Findings in African Pygmy Hedgehogs Admitted into a Portuguese Rehabilitation Center

**DOI:** 10.3390/ani12111361

**Published:** 2022-05-26

**Authors:** Gabriela Fernandes Silva, Alexandra Rêma, Sílvia Teixeira, Maria dos Anjos Pires, Marian Taulescu, Irina Amorim

**Affiliations:** 1 ICBAS-School of Medicine and Biomedical Sciences, Porto University, Rua de Jorge Viterbo Ferreira 228, 4050-313 Porto, Portugal; gafernandessilva@gmail.com (G.F.S.); alexandrarema@gmail.com (A.R.); silvia.goncalves.teixeira@gmail.com (S.T.); 2 Institute for Research and Innovation in Health (i3S), University of Porto, 4200-135 Porto, Portugal; 3 Institute of Molecular Pathology and Immunology of the University of Porto (IPATIMUP), Rua Júlio Amaral de Carvalho Nr. 45, 4200-804 Porto, Portugal; 4 Center of Animal and Veterinary Sciences, University of Trás-os-Montes e Alto Douro, 5000-811 Vila Real, Portugal; apires@utad.pt; 5 Pathology Department, Faculty of Veterinary Medicine, University of Agricultural Sciences and Veterinary Medicine, 400372 Cluj-Napoca, Romania; taulescumarian@yahoo.com; 6 Synevovet Laboratory, Industriilor Street, No. 25, Chiajna, Ilfov County, 077040 Chiajna, Romania

**Keywords:** African pygmy hedgehog, carcinoma, mycobacteriosis, pathologies, tumors, wobbly hedgehog syndrome

## Abstract

**Simple Summary:**

Hedgehogs are small mammals whose proximity to humans has been intensifying. However, knowledge on the possible predisposition to and transmission of diseases by these animals is limited; therefore, this study investigated the most relevant postmortem pathological features present in a group of six African pygmy hedgehogs admitted into a rehabilitation center. A significant and diverse set of pathologies with considerable value from the point of comparative pathology are described which highlight the importance of this small mammal in the eco-epidemiological context of zoonotic diseases and its important role in the concept of One Health, acting as “sentinels” of the environment in which they are found.

**Abstract:**

Most of the pathologies that affect hedgehogs are diagnosed postmortem; thus, it is essential to share knowledge between clinicians and pathologists in order to recognize predispositions to diseases and to establish adequate diagnostic and therapeutic plans. This study aimed to describe the most relevant postmortem pathological conditions in a group of six rescued African pygmy hedgehogs, performed over a period of four months. Hedgehogs were submitted to necropsy examinations and subsequent histopathological analyses. Microscopically, all the studied hedgehogs revealed alterations in one or more organ systems. Although a significant and diverse number of pathological conditions were obtained, this study focused on less common or more relevant pathologies found in African pygmy hedgehogs—namely, wobbly hedgehog syndrome, squamous cell carcinoma and mast cell tumors. Furthermore, this study constitutes the first report of *Mycobacterium* spp. in hedgehogs in Portugal, the second report of follicular thyroid carcinoma in an African pygmy hedgehog, the description of a lipoid pneumonia for the first time in this species and a lung adenocarcinoma—a pathology rarely reported in African pygmy hedgehogs.

## 1. Introduction

Hedgehogs are small, nocturnal and insectivorous mammal animals that belong to the order Eulipotyphla, family Erinaceidae [1,2]. Worldwide, 16 species of hedgehogs are recorded, but the most common is the African pygmy hedgehog (*Atelerix albiventris*) and the European hedgehog (*Erinaceus europaeus*) [3]. The African pygmy hedgehog, also known as the four-toed hedgehog, is widespread in West, Central and East Africa and can be found in a variety of habitats, such as grassland, scrub, savannah and suburban gardens [4,5]. Nowadays, its demand as a pet and its breeding in captivity have increased [5].

The knowledge of the diseases to which this species is possibly predisposed to is limited; nevertheless, several retrospective studies of disease incidence in pet hedgehogs have been published [6,7,8].

A 19-year retrospective study performed by Gardhouse and Eshar (2015) [6] assessed the occurrence of diseases in captive African pygmy hedgehogs, showing that the integumentary and gastrointestinal systems are the most affected. Acariasis or mite infestation and dermatophytosis are recurrent integumentary diseases of the African pygmy hedgehog [5,9]. The most frequent alimentary diseases include dental problems, enteritis caused by salmonella, candidiasis and cryptosporidiosis [9]. In wild hedgehogs, several species of nematodes, cestodes and protozoa have been identified; however, their significance in pet hedgehogs appears to be minimal [10].

Hepatic lipidosis and cardiomyopathy are common postmortem findings in African pygmy hedgehogs [9].

Additionally, wobbly hedgehog syndrome (WHS) is an insidious, progressive and incurable neurologic process that remains the leading cause of neurologic disease in these animals, affecting 10% of hedgehogs in North America [11].

Tumors are extremely common and represent the most widely reported pathological findings in the African pygmy hedgehog, which seems particularly prone to neoplasia [5,6,12]. In a recent study from 2012 to 2017, 100 African pygmy hedgehogs were examined, and neoplastic lesions were detected in 60%, with 74.6% of these tumors being classified as malignant [8]. Another study showed that neoplastic processes were the third most detected pathology in African pygmy hedgehogs, observed in 20.75% of cases, with 45.45% of these neoplasms being diagnosed as oral squamous cell carcinomas (SCC) [6]. Although more than one tumor type may be present, the most reported specific histological tumor types are mammary gland adenocarcinoma, lymphoma and oral SCC [13,14,15]. Neoplastic processes seem to essentially affect the integumentary, hemolymphatic, reproductive, digestive and endocrine systems. According to tumor histogenesis, epithelial tumors are more common, followed by round cell and mesenchymal or spindle cell tumors [8,14,15]. However, in the report of Okada et al. (2018) [8] mesenchymal tumors were more common than both epithelial and round cell tumors.

The African pygmy hedgehog has become popular as an exotic pet, but carries the risk of transmitting diseases with zoonotic potential [2]. Although rare in hedgehogs, some mycobacterial organisms are reported in these species. As prey animals and scavengers, hedgehogs can become infected with *Mycobacterium* spp. in a variety of ways, such as contamination of skin wounds and oral and aerosol transmission, which makes them an important element in the epidemiology of the infections caused by these bacteria [3,16].

Most of the pathologies that affect hedgehogs are diagnosed postmortem. Therefore, the aim of this study was to describe the most relevant macroscopic and microscopic findings identified during necropsy examinations of six rescued African pygmy hedgehogs, performed over a period of four months.

## 2. Materials and Methods

### 2.1. Study Design

Six rescued African pygmy hedgehogs (*Atelerix albiventris*) that died of natural causes or were euthanized were submitted for necropsy at the Veterinary Pathology Laboratory of ICBAS-UP in Porto, Portugal between February and June 2021. These animals were initially kept as pets, being later admitted for rehabilitation or disease recovery by the Portuguese association *Amigos Picudos*, located in Maia, Porto. It is important to note that no animal was euthanized for the purpose of this study. Further details about the study group, including species, sex, age and body weight are compiled in Table 1.

### 2.2. Histopathology and Histochemistry

The necropsy examination was performed and representative samples of tissues, with or without relevant macroscopic alterations, were collected and fixed in 10% neutral buffered formalin for microscopic examination. Samples taken from most animals included the lungs, digestive tract, kidneys and genital system, liver, spleen and heart. Given the presentation of neurological alterations, in cases 1 and 5, samples from the central nervous system (CNS) were also collected. Tissues were routinely processed and paraffin-embedded, and 2 μm thick serial sections were cut and stained with hematoxylin and eosin (HE). Appropriate histochemical stains were performed whenever necessary.

### 2.3. Immunohistochemistry

According to the individual case and aims, immunohistochemistry (IHC) was performed. A NovolinkTM Max-Polymer detection system (Novocastra, Newcastle, UK) was used according to the manufacturer’s instructions. Slides were incubated with a panel of specific antibodies, for which detailed characteristics are described in Table 2.

## 3. Results

Based on the macroscopic and histological findings, results were grouped according to the organ systems in which the most significant changes were noticed. Table 3 summarizes the histochemical stains performed and their results. In Table 4, pathological findings and respective diagnoses are grouped by systems.

### 3.1. Skin and Subcutaneous Tissue

**Figure 1:**
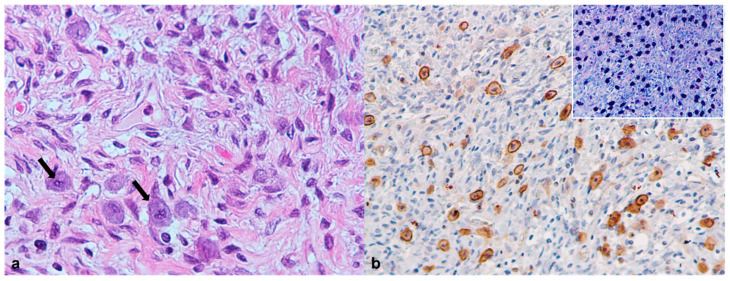
African pygmy hedgehog, subcutaneous tissue. Case 4, Mast cell tumor. (**a**) Presence of round cells supported by abundant collagenous stroma. Cells show high pleomorphism and variable amount of intracytoplasmic basophilic granules (arrows; HE, 100×); (**b**) C-kit immunostaining revealing strong and both membranous and cytoplasmic immunopositivity of neoplastic mast cells. Inset: neoplastic mast cells showing metachromatic cytoplasmic granules (Giemsa, 200×).

In case 4, microscopic analysis of a subcutaneous nodule from the neck region, measuring 2.0 cm in diameter, showed a neoplastic lesion with well-defined borders, consisting of round-to-oval cells with numerous basophilic and Giemsa positive intracytoplasmic granules, diagnosed as a subcutaneous mast cell tumor (MCT; Figure 1). Diffuse positive immunoreactivity for c-Kit confirmed this diagnosis (Figure 1b). The neoplastic cells showed moderate cellular pleomorphism and a low mitotic count (two mitotic figures per 10 high-power fields (HPFs)).

### 3.2. Respiratory Tract

In the present study, two animals were diagnosed with pneumonia. Histological examination allowed the detection of infectious granulomatous pneumonia (case 1) and lipoid pneumonia (case 4).

In case 1, macroscopic examination of the lungs revealed the presence of about 12 randomly disseminated yellowish-white elevated nodules, measuring approximately 1–5 mm in diameter. Histological examination revealed a chronic granulomatous pneumonia affecting 98% of the pulmonary parenchyma with the presence of multiple coalescent centers of caseous necrosis surrounded by macrophages, multinucleated giant cells (Langhans cells) and small lymphocytes. These were subjected to Ziehl–Neelsen staining, which allowed the identification of several intrahistiocytic acid-fast bacilli, potentially belonging to the genus *Mycobacterium* spp. Similar lesions were also detected in the kidney, liver and spleen.

**Figure 2:**
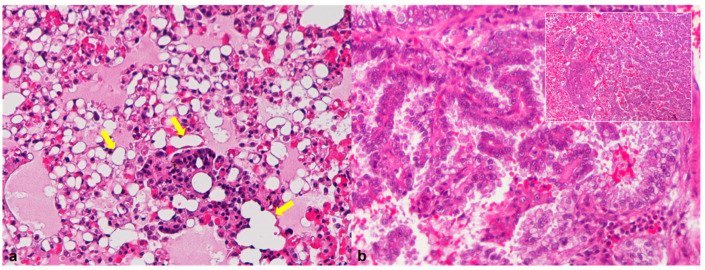
African pygmy hedgehog, lung. (**a**) Case 4, Lipoid pneumonia. Intra-alveolar and interstitial accumulation of lipid-laden macrophages (arrows; HE, 200×); (**b**) Case 6, Pulmonary adenocarcinoma. Population of neoplastic epithelial cells arranged in a papillary pattern (HE, 200×). Inset: presence of squamous differentiation in same areas of the neoplastic mass (HE, 100×).

Macroscopically, case 4 presented marked pulmonary alterations. Lungs showed compact, red and white marbled-color areas of compensatory emphysema at the periphery of the lobes. Microscopically, the alveoli were distended, with large numbers of macrophages containing numerous lipid droplets mixed with small lymphocytes, neutrophils and plasma cells (Figure 2a). Based on morphological features, lipoid pneumonia was diagnosed.

In case 6, the lung parenchyma was replaced by a large and multinodular neoplastic lesion, consisting of neoplastic epithelial cells arranged predominantly in a papillary pattern or, less often, in nests, exhibiting multiple foci of squamous differentiation. The neoplastic cells showed high cellular pleomorphism and moderate mitotic count (five mitotic figures per 10 HPFs). Thus, a diagnosis of a pulmonary adenocarcinoma was given (Figure 2b).

### 3.3. Digestive tract

#### 3.3.1. Oral Cavity

**Figure 3:**
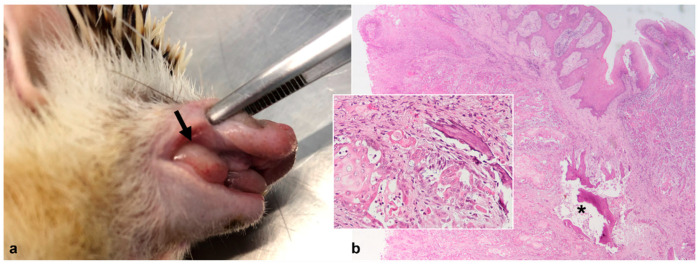
African pygmy hedgehog, oral cavity. Case 2, Squamous cell carcinoma. (**a**) Gross appearance of the oral mass in the right maxilla (arrow); (**b**) The mass consists of neoplastic squamous epithelial cells organized in nests. The neoplastic lesion infiltrates the underlying layers, including bone (*; HE, 20×). Inset: note the bone invasion by neoplastic cells and osteolysis (HE, 200×).

In case 2, an irregular, white-gray, firm mass, measuring 2.0 cm in diameter was detected, presumably arising from the gingiva, affecting the right maxilla and projecting into the palate (Figure 3a). Microscopically, an infiltrative neoplastic lesion consisting of squamous epithelial cells arranged in nests, islands and trabeculae and exhibiting central keratinization was observed (Figure 3b). The neoplastic cells showed high cellular pleomorphism and a low mitotic count (two mitotic figures per 10 HPFs). The neoplastic lesion was diagnosed as SCC.

In another case, an active chronic gingivostomatitis consisting of mucosal infiltration by neutrophils, plasma cells and lymphocytes was detected (case 3).

#### 3.3.2. Liver

Inflammatory changes compatible with a granulomatous and pyogranulomatous hepatitis were observed in cases 1 and 2, respectively. In case 4, severe and multifocal bile duct hyperplasia was identified. Additionally, in case 5, an interstitial hepatitis was also found.

In case 1, although macroscopically the liver did not show relevant alterations, the histological examination revealed granulomatous lesions similar to those described in the lungs. Ziehl–Neelsen staining was performed and showed acid-fast bacilli suggesting an infection with *Mycobacterium* spp.

In case 2, microscopic examination of the liver revealed multiple pyogranulomatous foci with neutrophils, eosinophils, lymphocytes, plasma cells and some multinucleated giant cells. Ziehl–Neelsen stain was negative.

**Figure 4:**
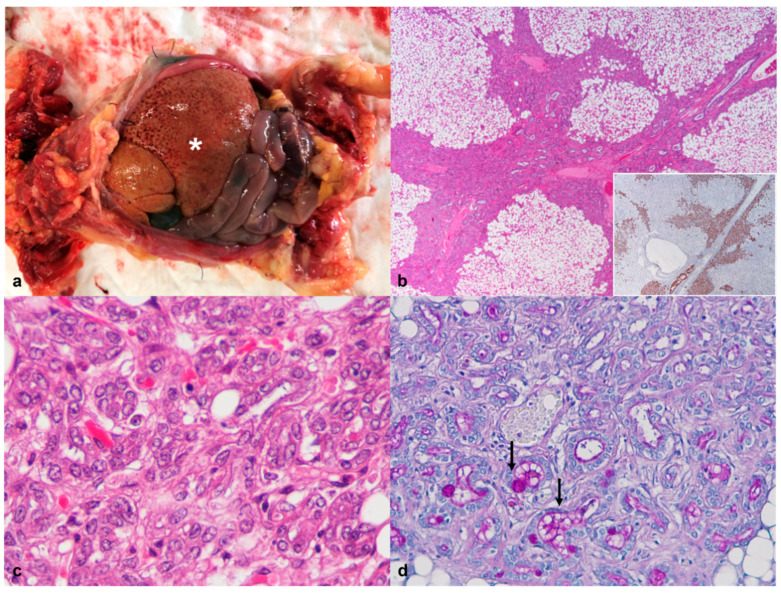
African pygmy hedgehog, liver. Case 4, Bile duct hyperplasia and hepatic steatosis. (**a**) Postmortem examination of the abdominal cavity demonstrating hepatomegaly (*); (**b**) Bile duct hyperplasia. Photomicrograph of the liver showing diffuse macrovesicular steatosis and severe proliferation of bile ducts associated with bridging fibrosis (HE, 20×). Inset: The bile duct epithelial cells showing cytokeratin (AE1/AE3) immunoreactivity, (20×); (**c**) The hyperplasic bile ducts are lined by cuboidal to polygonal epithelial cells with vesicular nuclei (HE, 400×); (**d**) Some of the tubular structures contain a PAS-positive material (arrows; PAS, 200×).

Hepatomegaly was a relevant gross finding in the case 4 (Figure 4a). Histologically, severe bile duct hyperplasia was observed. The liver architecture was multifocally effaced by large aggregates of variably sized bile ducts, lined by a single layer of cuboidal cells, surrounded by a moderate amount of connective tissue. The epithelial cells were positive for pan-cytokeratin (AE1/AE3) and displayed mild anisocytosis and anisokaryosis with homogenous or granular eosinophilic cytoplasm. Occasionally, the lumen of the bile ducts contained a PAS-positive amorphous material (Figure 4b–d).

The liver of case 5 presented necrotizing inflammation, hepatocellular swelling and vacuolar degeneration, and portal fibrosis; intrahepatic cholestasis and hemosiderosis were also observed.

Liver macrovesicular steatosis was also identified in cases 4 and 6. The lesions consisted of large intracytoplasmic vacuoles with well-defined borders and eccentric displacement of the nuclei. The presence of megakaryocytes suggesting an extramedullary hematopoiesis was a microscopic finding in the liver of two individuals (cases 4 and 5).

### 3.4. Urinary System

**Figure 5:**
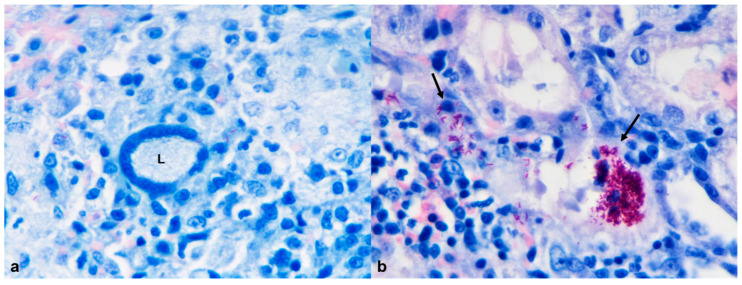
African pygmy hedgehog, kidney. Case 1, Mycobacterial interstitial granulomatous nephritis (Ziehl–Neelsen, 600×) (**a**) The inflammatory nodules contain multinucleated giant cell (Langhans type; L) admixed with mononuclear cells and rare neutrophils; (**b**) Presence of numerous acid-fast bacilli in the cytoplasm of macrophages (arrows).

In case 1, the kidney presented multiple round nodules measuring 1.0–2.0 mm in diameter. Histologically, granulomatous lesions similar to those observed in the lungs and liver of the same animal were identified (Figure 5).

### 3.5. Endocrine System

**Figure 6:**
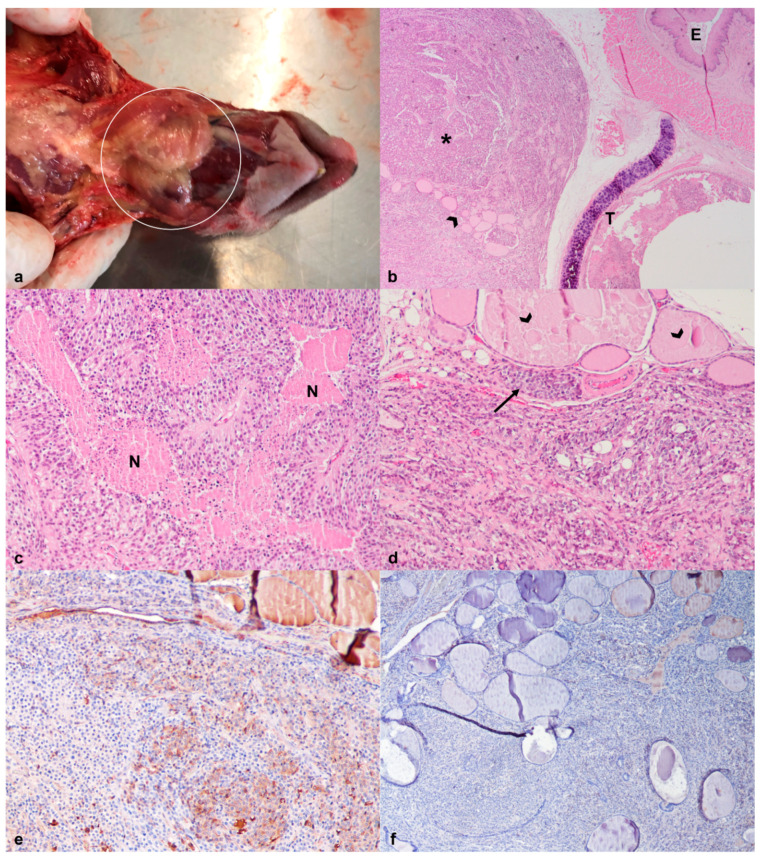
African pygmy hedgehog, thyroid gland. Case 2, Thyroid carcinoma. (**a**) Gross appearance of the bilobed cervical mass (circle); (**b**) The lesion is composed of neoplastic epithelial cells (*), forming solid structures separated by a fine fibrovascular stroma, and compressing the esophagus (E) and trachea (T). The remaining thyroid gland follicles are visible, particularly at the periphery of the neoplastic mass (arrowhead)( HE, 20×); (**c**) Multifocal areas of intratumoral necrosis (N; HE, 100×); (**d**) Note the foci of extracapsular tumor growth (arrow) and few remaining thyroid follicles (arrowhead; HE, 100×); (**e**) Considerable percentage of neoplastic cells present weak to moderate thyroglobulin-immunopositivity (100×); (**f**) Low percentage of neoplastic cells present weak immunoreactivity to synaptophysin (100×).

In case 2, the macroscopic examination of the thyroid gland revealed a bilobed cervical mass measuring 1.5 cm in diameter, with well-defined borders (Figure 6a). Subsequent histological analysis showed that both thyroid lobes were replaced by a multinodular neoplastic lesion compatible with a solid carcinoma. The lesion was highly cellular and composed of neoplastic epithelial cells, forming solid nests separated by a scant fibrovascular stroma (Figure 6b–d). The neoplastic cells were cuboidal to polygonal and exhibited a moderate amount of finely vacuolated acidophilic cytoplasm and variably distinct cellular borders. The nuclei were vesicular, basal to paracentral, with 1–2 basophilic prominent nucleoli. The number of mitotic figures varied from 0 to 1 per HPF. Congo red staining was evaluated through polarized light, but no amyloid-like material was detected. The results of the immunohistochemical study performed in this case are summarized in Table 5.

### 3.6. Lymphatic System

#### Spleen

Granulomatous mycobacterial splenitis was identified in case 1. Histological examination of the spleen revealed coalescent centers of caseous necrosis and granulomatous inflammation consisting of macrophages, Langhans cells and small lymphocytes, associated with the presence of intrahistiocytic acid-fast bacilli.

**Figure 7:**
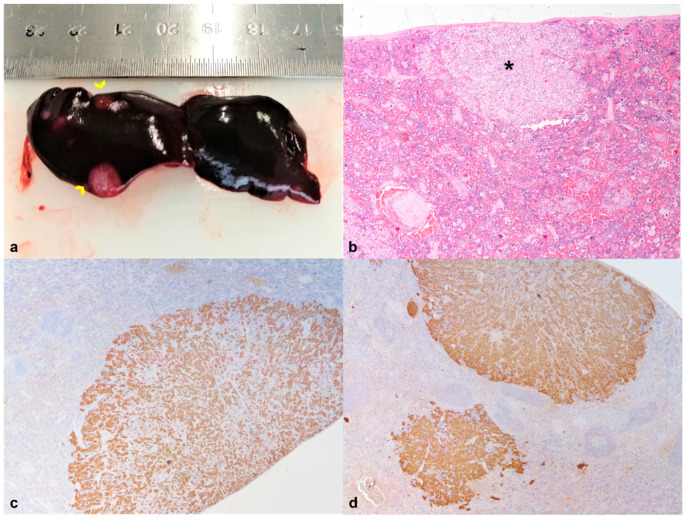
African pygmy hedgehog, spleen. Case 2, Metastases of a thyroid carcinoma. (**a**) Presence of several white-gray nodular masses, measuring 1.0–5.0 mm diameter (yellow arrows). (**b**) Well-demarcated nodular lesion (*) composed of neoplastic epithelial cells arranged in a solid pattern (HE, 20×); (**c**,**d**) The great majority of neoplastic cells showed strong immunoreactivity for pan-cytokeratins (AE1/AE3) and thyroglobulin (20×).

In case 2, five white-gray well-circumscribed nodular masses measuring between 1.0–5.0 mm in diameter were macroscopically identified in the spleen (Figure 7a). Histologically, a diagnosis of carcinoma was made (Figure 7b). IHC was performed for pan-cytokeratins (AE1/AE3; Figure 7c) and thyroglobulin (Figure 7d), revealing strong and diffuse immunopositivity of the neoplastic cells. Based on the histological and immunohistochemical findings, a metastatic thyroid gland carcinoma in the spleen was confirmed.

Presence of numerous megakaryocytes, suggesting an extramedullary hematopoiesis, was a relatively common microscopic finding in the spleen of all the studied animals.

### 3.7. Central Nervous System

**Figure 8:**
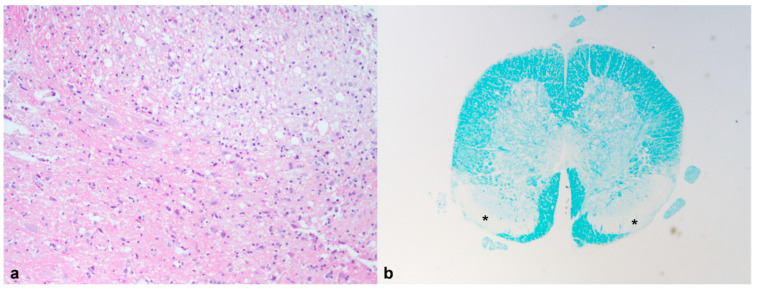
African pygmy hedgehog, Medulla oblongata. Case 1, Wobbly hedgehog syndrome. (**a**) Note the white matter spongiosis in the medulla oblongata (HE, 100×); (**b**) Bilateral pale areas (*), representing myelin loss, in the lower tracts of the medulla oblongata (Luxol fast blue, 20×).

Case 1 was diagnosed with WHS, in which histological examination of the brain and spinal cord demonstrated severe neurodegenerative processes. Multifocal areas of myelin degeneration and loss, accompanied by neuronal degeneration, microgliosis and spongiosis were observed in the cerebellum and medulla oblongata (Figure 8a). Luxol fast blue stain showing myelin loss in the ventral regions of the medulla oblongata (Figure 8b). Status spongiosus was more prominent in the white matter and was characterized by the presence of numerous variably sized round clear spaces.

Case 5 also showed histological features of CNS neuronal degeneration, and the Luxol fast blue stain confirmed the myelin loss—however, this was not sufficient to meet the criteria for diagnosis with WHS.

### 3.8. Reproductive Tract

Only one animal (case 4) was diagnosed with reproductive tract neoplasia. A multinodular ovarian mass measuring 4.0 × 4.0 × 1.5 cm was identified. The histological evaluation showed a well-circumscribed neoplastic lesion composed of numerous spindle cells (fibroblasts) arranged in short bundles, with moderate cellular pleomorphism and low mitotic count (1 mitosis/10HPF). Masson’s trichrome stain confirmed the presence of collagen fibers in the connective stroma of the tumor. A definitive diagnosis of an ovarian fibroma was made.

## 4. Discussion

The present study aimed to analyze the macro- and microscopic findings of a collection of six African pygmy hedgehogs.

These animals’ high propensity for developing neoplasia seems to be consistent worldwide, and this study is in agreement with these results. It is speculated that increased lifespan in captivity leads to a higher incidence of neoplastic conditions in this species [7].

As with other small mammals, extramedullary hematopoiesis of indeterminate cause is frequent in hedgehogs—although, in some cases, it may be related to anemia and systemic infections [17]. It usually involves the spleen; however, in this study, histological images compatible with extramedullary hematopoiesis were also found in the liver.

Concomitant diseases are routinely found in postmortem examinations of African pygmy hedgehogs [7], which is in line with the results of this study. Despite the presence of several pathologies affecting multiple organ systems, these are often incidental findings. This reinforces that they are strong and resilient animals, often not showing clinical signs of disease.

In the discussion section of this study, uncommon or less common pathologies reported to date in these species will be critically and thoroughly addressed—namely, cases 1, 2, 4 and 6.

### 4.1. Case 1

Case 1 was represented by a two-year-old African pygmy hedgehog which essentially had a neurological clinical history. Histological examination of multiple organs revealed a systemic disease compatible with *Mycobacterium* spp. infection, mostly disseminated through the lungs, liver, kidneys and spleen, and a concomitant picture of WHS.

There is only single report of *Mycobacterium tuberculosis* var. *bovis* that causes typical tuberculosis [18] and of *Mycobaterium avium* ssp. *paratuberculosis*, a causative agent of paratuberculosis in ruminants—both in European hedgehogs [19]. Although *M. bovis* mainly affects the respiratory tract, small granulomas can also develop in the liver, kidneys and spleen. In 2014, a study conducted to determine the prevalence of *M. bovis* in Portugal didn’t reveal the infection in hedgehogs [20].

*Mycobacterium marinum* is classified as an atypical non-tuberculous mycobacterium (NTM) and there are three reports of this mycobacteriosis in hedgehogs: a cutaneous form has been described in an African pygmy hedgehog [16] and two cases of systemic infection in a European and in an African pygmy hedgehog [21,22]. *M. marinum* is ubiquitous in aquatic environments and causes a chronic progressive disease in various freshwater and saltwater fishes. In humans, it also causes cutaneous infection (“fish tank granuloma”) due to traumatic injuries associated with contaminated fish, fish tanks or swimming pools. However, severe manifestations as a disseminated infection have been reported, particularly in immunosuppressed patients [21]. In the case reported by Tappe et al. (1983) [22], the European hedgehog became infected with *M. marinum* through contact with water from a nearby fish tank located in the same pet store.

Molecular analysis would be indispensable for specific etiologic agent species identification. In the present case, DNA extraction was obtained from paraffin tissue sections and real-time PCR was performed at the Institute for Research and Innovation in Health (i3S), allowing the exclusion of *Mycobacterium tuberculosis* and *Mycobacterium africanum* (data not shown).

Although WHS can occur at any age, it most frequently affects hedgehogs less than two years of age, as occurred in this case [23]. Additionally, the neurological clinical signs displayed by this animal (ataxia and lameness of the hind limbs), along with the histological alterations detected in the CNS were compatible with WHS.

The bilateral and symmetrical vacuolization of the CNS white matter, associated with myelin degeneration and loss, were confirmed by Luxol fast blue stain, which has proven to be an excellent method for aiding in this diagnosis. In this case, the neurodegenerative processes were more pronounced in the cerebellum and medulla oblongata. These alterations can be the cause of the lack of the animal’s mobility and can further explain the cutaneous wound found in its right forelimb—a common finding in hedgehogs with WHS, as the result of the animal dragging its limbs due to the inability to fully extend them and the loss of their full range of motion [23].

Hepatic and renal pathologies seem to be incidental findings in hedgehogs with WHS [23]. In this case, these organs were severely affected by mycobacterial granulomas. Some authors claim that WHS lesions resemble hepatic and renal encephalopathy syndromes [11]. Thus, a potential link between the granulomatous lesions, liver and kidney failure that could lead to encephalopathy and to histological pictures similar to WHS must also be considered.

The lack of knowledge concerning the specific *Mycobacterium* species present in these lesions prevents further inferences regarding the source and the pathogenic route of this infection. This hedgehog was kept as a pet, which suggests a controlled environment. Nevertheless, the ulcerated skin identified in the right forelimb may also represent a potential bacteria entrance point, with consequent dissemination to other organs. However, in transcutaneous infection, extensive necrosis of the inoculated skin areas is usually observed, with secondary edema of the respective limb and enlargement of the regional lymph nodes [16,21]—which was not observed in this case. On the other hand, WHS is a common condition in African pygmy hedgehogs and a genetic predisposition to this disease is recognized in these animals [11]. Thus, WHS disease progression may have led, to a certain point and extent, to an immunodepressive state that favored a secondary *Mycobacterium* spp. infection and dissemination.

### 4.2. Case 2

The patient was an African pygmy hedgehog more than three years of age diagnosed with an oral SCC and a thyroid carcinoma with splenic metastases. Although both neoplasms are relatively common in hedgehogs, what makes this case interesting is the concomitant presence of two distinct malignant neoplasms. The prevalence and clinical significance of thyroid masses vary widely between species. Humans usually have benign thyroid nodules. Likewise, they are also relatively common in older cats—most consisting of functional adenomatous hyperplasia. In contrast, thyroid neoplasms are rare in dogs and, when seen, they are predominantly malignant [24].

Thyroid carcinomas are usually large, solid, firm, irregular and non-painful masses that commonly invade adjacent structures [24,25,26]. Usually, these tumors are identified close to the typical location of the normal thyroid and may be unilateral or, as in this case, bilateral. Case-reports describing these neoplasms in hedgehogs often report associated clinical signs such as dysphagia, weight loss, polydipsia and tetraparesis [25,26]. In this case, the animal presented with severe tachypnea, which, given the large size of the mass, could be due to the compression of the thyroid adjacent structures. Distant metastases are common in the lungs and regional lymph nodes, but may also affect the jugular vein, liver, adrenal gland, kidneys, heart base, spleen, bone and bone marrow, prostate gland, brain, skeleton and spinal cord [27].

Commonly, thyroid carcinomas originate from two distinct endocrine cell types [27]. Thyroid tumors of follicular cell origins arise from epithelial cells that line the colloid follicles. These cells are able to concentrate iodine and are involved in thyroid hormone production [24]. Depending on their pattern of growth, thyroid tumors may be classified into follicular, compact (solid), papillary, compact-follicular, or undifferentiated (anaplastic) carcinomas [27]. In addition, medullary thyroid carcinomas arise from the parafollicular C-cells, which produce calcitonin and are part of the amine precursor uptake decarboxylation (APUD) system [27]. Most thyroid tumors in humans, dogs and cats arise from the follicular epithelium. Medullary thyroid tumors are relatively rare, occurring in less than 10% of thyroid tumors in these species [24]. In hedgehogs, the literature on and reported cases of thyroid carcinomas are scarce. However, follicular adenocarcinoma and C-cell carcinoma have been previously reported in African pygmy hedgehogs [15,25,26].

In this case, the histological image was compatible with a solid carcinoma originating from thyroid follicular cells. However, the distinction between follicular or medullary thyroid carcinoma through conventional microscopy is not clear [28]. Thyroid tumors that arise from parafollicular C-cells often have a compact cell growth pattern, making it difficult to distinguish solid follicular tumors with similar histological appearances by routine light microscopy alone [27]. The presence of amyloid interspersed with the tumor cells is a variable finding among species affected with C-cell carcinoma. However, in hedgehogs, amyloid material is not a major feature of this tumor [26]—as in the present case, where no amyloid was detected using Congo red staining through polarized light.

Thyroid tumors derived from follicular cells routinely stain positively for thyroglobulin. In contrast, C-cell tumors have demonstrated strong immunoreactivity to calcitonin and more variable staining for synaptophysin [24]. According to the immunohistochemical study performed, the great majority of the neoplastic cells were immunopositive for thyroglobulin. Labeling for synaptophysin and TPO was weak and only a few tumor cells showed immunopositivity for synaptophysin. Nevertheless, the controls for NSE and synaptophysin immunomarkers were from canine tissue, requiring a period of validation and optimization—so the interpretation of results must be cautious.

Given the positive results for these cells’ immunomarkers, along with the weak or non-existent immunoreactivity for the parafollicular cell markers, the results of this immunohistochemical panel suggest that the neoplastic lesion is of a presumptive follicular origin.

The epithelial immunophenotype of the neoplastic cells that constitute the nodular lesions found in the splenic nodules, further confirmed through AE1/AE3 immunopositivity, make it plausible to hypothesize that they constitute metastases of a primary neoplasm—which in this specific case, could be related to either SCC or the thyroid carcinoma. As for SCC, the spleen is not a common site of metastases, which usually tends to metastasize to the lungs [17]. Additionally, the thyroglobulin-immunopositivity of the splenic neoplastic cells reinforced a thyroid histogenesis.

Pesticide and environmental contaminants have been reported as endocrine-disrupting chemicals, provoking thyroid-disrupting effects in several vertebrate species. It is known that some chemicals can interfere with the regulation of thyroid hormones by the hypothalamus and pituitary gland, affecting their synthesis [29]. Given the existence of environmentally contaminated areas in Portugal [29], this animal may have been at risk of exposure to products with negative effects on its health; however, we do not have enough data to confirm this. Thus, further investigations should be carried out to assess to what extent thyroid pathologies can predict the level of environmental contamination of the habitat of this species.

### 4.3. Case 4

This case represents a five-year-old obese African pygmy hedgehog diagnosed with lipoid pneumonia which, to date, is thought to be the first report of this condition in hedgehogs. The same animal presented with other pathologies: a severe bile duct hyperplasia, a subcutaneous MCT and an ovarian fibroma (minor emphasis will be provided to the latter, as it remains a common and incidental finding).

Lipoid pneumonia is an uncommon, non-infectious, inflammatory lung disease histologically characterized by intra-alveolar lipid and lipid-laden macrophages in the alveoli [30,31]. Consolidation of the lung parenchyma, accumulation of acellular acidophilic substance and fibrin exudation were among the histological findings in this animal, which is consistent with previous reports of this pathology in other species [30].

Depending on the lipid source, this condition is classified as exogenous or endogenous lipoid pneumonia (EnLP). The former has been widely described in human and veterinary medicine and is caused by a chronic foreign body reaction to fatty substances in the alveoli, after the inhalation or aspiration of laxative mineral oils [31]. Although the pathogenic mechanism of EnLP is not completely understood, it is probably related to pneumocyte injury, leading to alveolar lipid deposition [30,31].

EnLP has been associated with infectious agents (bacteria and parasites), obstructive pulmonary diseases and reduced airway clearance. In addition, it has been associated with atherosclerosis and hepatopathy [30]. In this case, bacterial and parasitic infections were considered less likely, since organisms were not microscopically observed and the histochemical Grocott, PAS and Gram stains performed were also negative. Less commonly, other processes may be involved in the appearance of endogenous lipids in the lung: fat embolism, pulmonary alveolar proteinosis, Wegener’s granulomatosis or lipid storage diseases [32]. In human medicine, EnLP is known to be associated with pulmonary neoplasia. Indeed, both concomitant pathologies have already been described in cats [31,33].

The liver is a major regulator of body metabolism and performs several critical functions, playing a central role in lipid metabolism—namely, in the synthesis, storage and degradation of lipids [34]. Likewise, macrophages help to clear lipids from the lungs. Thus, the mechanisms involved in lipoid pneumonia may imply an inflammatory response to increased lipid uptake by alveolar macrophages [32]. In this way, perhaps a dysregulation in lipid metabolism derived from hepatic dysfunction may be at the origin of the pneumonia found in this case. On other hand, we cannot exclude the hypothesis that the primordial pathological process is an alteration in lipid metabolism, which would justify the lipid vacuoles observed in the lung, the hepatic steatosis and the bile duct hyperplasia presented in this animal.

MCTs in hedgehogs are rare in the veterinary literature, with only four previous reports [35,36]. In this species, different kinds of mast cell diseases are reported: (1) the cutaneous form is classified into localized and diffuse cutaneous mastocytosis and (2) systemic mastocytosis is characterized by the proliferation of mast cells in the internal organs [35]. The cutaneous form often affects the head, neck and axillary region. In hedgehogs, MCTs can metastasize into regional lymph nodes [17]. There is also a case in which mast cells were reported in the spleen; however, in insectivorous animals, their presence is quite common in this organ [36]. In the case of this animal, no metastasis was observed.

Contrary to dogs, there are no specific standards for determining the malignancy grade of hedgehogs’ MCTs. Thus, to evaluate the MCT malignancy grade, the classical criteria of cellular morphology, number of cytoplasmic granules, number of cell nuclei, number of mitotic figures per HPF and infiltration of deeper tissues are considered relevant points [35].

### 4.4. Case 6

A five-year-old African pygmy hedgehog was diagnosed with a pulmonary adenocarcinoma, a malignant epithelial tumor that develops after neoplastic transformation of pneumocytes or bronchial epithelium [37].

In veterinary medicine, lung adenocarcinoma is usually a chronic and slowly progressive disease, and clinical signs are usually observed in cases of tumors of considerable size. Animals with lung tumors may experience progressive emaciation, weight loss and impairment of normal respiratory function due to excessive production of fluid in the lungs [37]. It can have a non-infectious or infectious etiology. Non-infectious causes are non-transferable and have a low incidence in animals. Infectious causes are essentially associated with the Jaagsiekte Sheep Retrovirus, causing a lung tumor known as Ovine Pulmonary Adenocarcinoma [37].

According to the World Health Organization (WHO) classification, microscopically, these tumors show various growth patterns (acinar, papillary, bronchioloalveolar, solid with mucin production or a mixed pattern) and is classified into nine types: bronchial gland carcinoma, squamous cell carcinoma, adenocarcinoma, adenosquamous carcinoma, small-cell carcinoma, large-cell carcinoma, neuroendocrine tumor, pulmonary blastoma and combined carcinoma [37,38,39].

Squamous metaplasia of the lung glandular epithelium may precede the development of squamous cell carcinoma of the lung; however, metaplasia alone is not sufficient to diagnose adenosquamous carcinoma [38,39].

In hedgehogs, primary lung neoplasms are rarely reported. Indeed, only two cases of lung adenocarcinoma [40]—a bronchoalveolar carcinoma and lung squamous cell carcinoma [14]—have been documented in African pygmy hedgehogs.

## 5. Conclusions

This study provided new insights into pathological diseases that can be found in postmortem examinations of hedgehogs. A significant and diversified number of pathological conditions was obtained, posing considerable value from the comparative pathology point of view. The variety of lesions and diseases highlight the importance of this small mammal in the eco-epidemiological context of disease, considering its potential to carry zoonotic diseases and its relevant role in the One Health concept.

To date, this constitutes the first report of *Mycobacterium* spp. in hedgehogs in Portugal. Although already reported, this remains only the second report of thyroid carcinoma in hedgehogs. In addition, this study describes a lipoid pneumonia for the first time in this species and a lung adenocarcinoma—a pathology rarely reported in African pygmy hedgehogs.

Therefore, for the correct diagnosis of pathologies in hedgehogs, it is essential to optimize and validate the complementary diagnostic methods commonly used in pathology laboratories, since the existing laboratory tools and protocols are not specifically directed to these species.

## Figures and Tables

**Table 1:** Detailed information about the animals included in the study.

Case	Species	Sex	Age (Years)	Body Weight (g)	Clinical History
1	African pygmy hedgehog (*Atelerix albiventris*)	F	2	245	Ataxia and lameness of the hind limbs (lasting six months) Malnutrition Skin wound in the right forelimb
2	African pygmy hedgehog (*Atelerix albiventris*)	F	>3	336	Severe tachypnea Mass in the oral cavity Subcutaneous mass in the neck region suspected of lymphadenomegaly
3	African pygmy hedgehog (*Atelerix albiventris*)	F	--	--	Nodule in the oral cavity
4	African pygmy hedgehog (*Atelerix albiventris*)	F	5	625	Breathing difficulty Obesity (body condition 9 (score 1–9))
5	African pygmy hedgehog (*Atelerix albiventris*)	M	--	240	Ataxia and nonspecific neurological symptomatology
6	African pygmy hedgehog (*Atelerix albiventris*)	M	5	--	Breathing difficulties

M: male; F: female; --: unknown.

**Table 2:** Details about the protocol and antibodies used for immunohistochemical study.

Antibody	Clone	Supplier	Dilution	Antigen UM	IT	Positive Control	Case
Thyroglobulin	Polyclonal	Dako, Denmark	1:2000	RS/WB	ON	Hedgehog normal thyroid	2
Thyroperoxidase (TPO)	Monoclonal (MoAb47)	Abcam, UK	1:20	EDTA pH = 9/WB	ON	Hedgehog normal thyroid	2
Neuron-specific enolase (NSE)	Monoclonal (VI-H14)	Imgenex, USA	1:500	RS/WB	ON	Canine normal pancreas	2
Cytokeratins (AE1/AE3)	Polyclonal	Thermo, USA	1:300	RS/WB	ON	Canine uterus and internal control	2, 4
c-Kit (CD117)	Polyclonal	Dako, Denmark	1:450	RS/WB	ON	Canine mast cell tumor	4
Synaptophysin	Monoclonal (SP11)	Thermo, USA	1:150	RS/WB	ON	Canine normal pancreas	2

Antigen UM: antigen unmasking; RS: Target Retrieval solution (Dako, Santa Clara, CA, USA); WB: water bath (100 °C; 20 min); IT: incubation time; ON: overnight.

**Table 3:** Commonly used special stains.

Case	Histochemical Stain	Structure Identification	Tissue	Result
1	Ziehl–Neelsen	*Mycobacterium* spp. (acid-fast bacilli)	Lung; spleen; liver; kidney	+
	Luxol fast blue	Myelin/loss of myelin	CNS	+
2	Ziehl–Neelsen	*Mycobacterium* spp. (acid-fast bacilli)	Liver	−
	Congo red	Amyloid	Cervical mass	−
4	Grocott methenamine silver	Fungal organisms	Lung	−
	Periodic acid–Schiff	Fungal hyphae	Lung	−
		Glycogen	Liver	+
	Gram stain	Bacteria	Lung	−
	Giemsa stain	Mast cells	Cervical nodule	+
	Masson’s trichrome stain	Connective tissue (fibrosis)	Ovary, liver	+
5	Luxol fast blue	Myelin/loss of myelin	CNS	+
	Perl’s Prussian Blue	Hemosiderin	Liver	+
	Hall’s Bilirubin Stain	Bilirubin	Liver	+

+: positive; −: negative

**Table 4:** Main pathological findings grouped by systems.

Case	Respiratory System	Digestive Tract and Liver	Urinary/Reproductive System	Endocrine System	Lymphatic System	Central Nervous System	Skin and Subcutaneous Tissue
1	Granulomatous pneumonia (*Mycobacterium* spp.)	Granulomatous hepatitis (*Mycobacterium* spp.)	Granulomatous nephritis (*Mycobacterium* spp.)		Granulomatous splenitis (*Mycobacterium* spp.)	Myelin degeneration Spongiosis and microgliosis	Ulcerative and necrotizing dermatitis
2		Granulomatous hepatitis Oral squamous cell carcinoma		Thyroid Carcinoma	Metastases of thyroid carcinoma in the spleen		
3		Chronic lymphoplasmacytic and ulcerative gingivostomatitis					
4	Lipoid pneumonia	Liver steatosis Hepatic extramedullary hematopoiesis Bile duct hyperplasia	Chronic interstitial nephritis Ovarian fibroma				Subcutaneous mast cell tumor
5		Acute necrotizing hepatitis Cholestasis; Hemosiderosis Hepatic extramedullary hematopoiesis				Myelin degeneration	
6	Pulmonary adenocarcinoma	Liver steatosis					

**Table 5:** The immunohistochemical analysis of the thyroid gland mass (Case 2).

Immunomarker	Results
Thyroglobulin	Mild to moderate cytoplasmic immunostaining in 70% of the neoplastic cell population (Figure 6e).
TPO	Weak and diffuse immunopositivity affecting 50–75% of the neoplastic cells.
Synaptophysin	Weak immunostaining in 30% of the neoplastic cells, more evident in the cells adjacent to the outer capsule (Figure 6f).
NSE	Intense and diffuse cytoplasmic immunostaining of more than 75% of the neoplastic cells.

## Data Availability

The data presented in this study are available in this article.
